# You Are Not Alone: A Medical Student Lead, Near-Peer Program to Support Preclinical Medical Students Remediating Examinations

**DOI:** 10.1007/s40670-026-02880-8

**Published:** 2026-09-15

**Authors:** Francisca Kolibar, Mehak Chawla, Gavin Wu, Anne Hinze, Christopher R. Pierson

**Affiliations:** 1 Department of Obstetrics and Gynecology, University Hospitals Cleveland Medical Center, Cleveland, OH 44106 USA; 2 The Ohio State University College of Medicine, Columbus, OH 43210 USA; 3 Department of Biomedical Education and Anatomy, Division of Anatomy, The Ohio State University, Columbus, OH 43210 USA; 4 Department of Pathology, The Ohio State University, Columbus, OH 43210 USA; 5 Department of Pathology and Laboratory Medicine, Nationwide Children’s Hospital, J0359 700 Children’s Drive, Columbus, OH 43205 USA

**Keywords:** Near-peer, Peer support, Preclinical, Undergraduate Medical Education

## Abstract

You Are Not Alone (YANA) is a student-faculty partnership that supports preclinical medical students in academic difficulty who need to remediate a final summative assessment. YANA members are students who previously remediated examinations and confidentially engage in shared storytelling and empathic listening to meet the student in difficulty where they are. The goals of YANA are to ameliorate the isolation these students commonly feel and to reframe remediation as part of the learning process. Here we describe YANA’s workflow and impact. We believe other schools could form such a group to enhance student support as a complement to school-based resources.

**Supplementary Information:**

The online version contains supplementary material available at https://doi.org/10.1007/s40670-026-02880-8.

## Background

Medical students are high achievers yet occasionally some struggle academically and need to remediate an examination. The remediation process varies among institutions, but commonly includes additional or alternative academic support, which may lack individualization, and be viewed as unable to meet a student’s specific needs [1]. Academic difficulty is often unexpected for a once thriving student who is forced to re-examine their self-perception and self-worth [2]. A review of the impact academic difficulties have on students indicated a need for individualized remediation strategies with educators and students working together to build trust [1]. A holistic remediation program includes cognitive and emotional approaches to learner support, but the latter is often underemphasized or is focused on reducing negatively valenced emotions [3]. Providing the remediating learner a supportive coaching relationship is a potential means to afford greater attention to fostering positive emotions [3].

Students approach peers more easily than faculty and academic difficulty is a common reason students seek peer support [1, 4, 5]. Peer-assisted learning has recognized value in helping students improve medical knowledge and well-being [6]. Others suggest having a means for a near-peer to share their experiences with a student in difficulty is beneficial [2]. Peer support can meet a student in their moment of need using a personal, shared-story approach, so peer-led, faculty-supported efforts hold promise in helping learners overcome challenges while promoting well being [7].

Given the preclinical curriculum’s rapid pace, students may adopt passive learning strategies to remediate promptly and avoid falling behind [8]. The impact of the experience often lingers, and students may feel like the process was untailored to their individual learning needs or even punitive [1]. Our college offers academic support and personal counseling to all remediating students, but we sought to add an individualized, student-centered approach, so we piloted You Are Not Alone (YANA), a student-led near-peer support group for remediating preclinical students. YANA is a group of empathetic near-peers, providing non-judgmental, confidential support, to de-stigmatize poor examination performance. YANA’s purpose is to provide students in difficulty personalized support using shared experience to address the isolation remediating students feel and reframe remediation as part of the learning processes. Here we describe YANA’s structure and effectiveness.

## Activity

Our preclinical curriculum is 18 months long and is organized into eight system-based blocks directed by doctorate level faculty. Medical knowledge is assessed in each block using a final summative examination and, in the five blocks covering anatomy, a laboratory practical examination. Students scoring below the passing threshold of 70% are required to remediate by passing a different version of the examination that is of comparable difficulty and covers the same learning objectives. On average, only 37 students in each class of 209 will need to remediate a block examination, which may contribute to feelings of isolation.

YANA members are empathetic near-peer medical students in second year or above who previously remediated an examination. Candidate members are selected following an interview process and are trained in formal meetings with YANA leaders to communicate program goals.

YANA’s process begins with the learning specialist (AH) emailing each student scoring below 70%, inviting them to a meeting during which the student is informed of YANA and provided a GroupMe link to contact YANA (Supplemental Information 1) [9]. A GroupMe is created for each group of students who are remediating a given block examination to foster connection among the students and YANA members. Initially the GroupMe is used to address common questions and allow each student to determine what support they need, in time it is used weekly to check student progress. Because GroupMe lists the names of all group members, the learning specialist also offers the opportunity to be connected directly with a YANA member if a student prefers anonymity. YANA members also meet with remediating students individually or in groups upon request to listen and provide encouragement with non-judgmental support to de-stigmatize the low examination score. These conversations explore feelings about remediation, concerns of falling behind, and tips to prepare for remediation. These conversations are based on shared experience and explore self-perception and self-value, while helping reframe remediation as a natural part of the learning process that may signal a need to change study strategies. Interacting with others who are remediating makes remediation a shared experience, fosters connection and reduces isolation. YANA members provide no personal or academic counseling but know who to contact for such support. Remediating students may be reluctant to seek help, so YANA works with faculty to plan faculty-led review sessions for interested students. Working with YANA is optional. Every student has the same access to the college’s support resources whether they work with YANA or not. To increase awareness among the class, YANA’s purpose is presented to the student body at the begining of each block. To build faculty support, YANA presents at academic program committee meetings, and its faculty advisor co-directs the preclinical curriculum.

The perceived effectiveness of YANA was evaluated using a voluntary, anonymous REDCap survey that was sent to remediating students (Supplemental Information 2) [10]. Survey items assessed participant experiences and YANA’s overall effectiveness.

## Results and Discussion

An email containing the survey link was sent to all 147 remediating students from the classes of 2024, 2025, 2026 and 2027. A reminder email was sent two weeks later, and the survey closed two weeks afterwards. The total response rate was 28% (n = 41). Ten respondants did not engage with YANA either to ensure anonymity (6 respondents), or due to the sense they could remediate independently (4 respondents). Of the 31 students who engaged YANA, 65% did so via the GroupMe and 16% in private meetings with YANA members, while 19% of participants engaged YANA by a combination of private meetings and the GroupMe (Table 1).

**Table 1:** How students engaged with YANA

Survey question	%	N
Engaged with YANA using the GroupMe	65%	20
Engaged with YANA by talking to a member of the group privately	16%	5
Engaged with YANA by meeting with a member and by using the GroupMe	19%	6

Abbreviations: % and N; percent and number of the 31 survey respondents who engaged with YANA, respectively

**Fig. 1:**
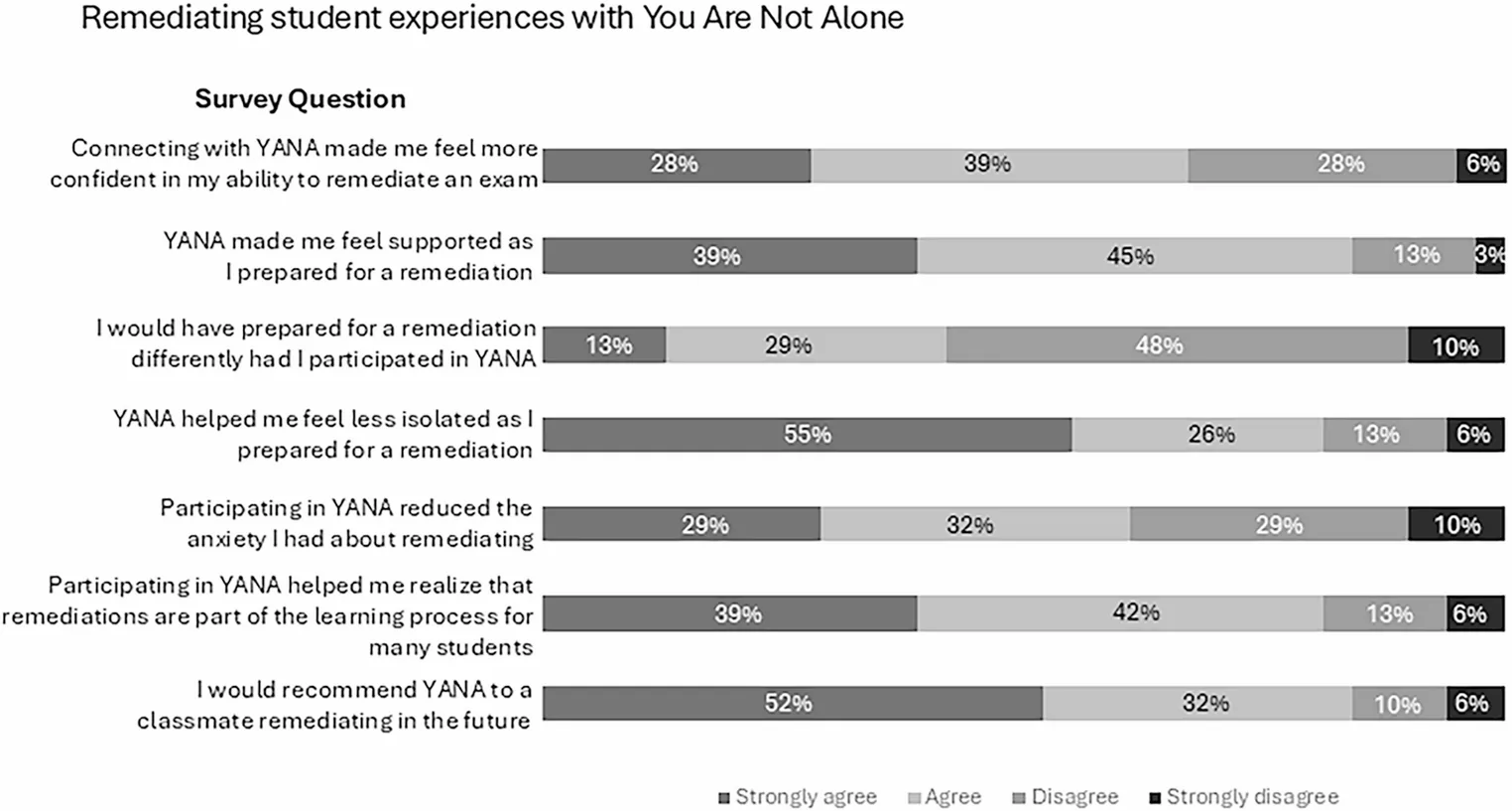
Survey results for items asking participants about their experiences working with YANA. Likert scale ranged from “strongly agree” to “strongly disagree”. Data reported as percent of respondents who engaged YANA. Figure created using Microsoft PowerPoint

**Fig. 2:**
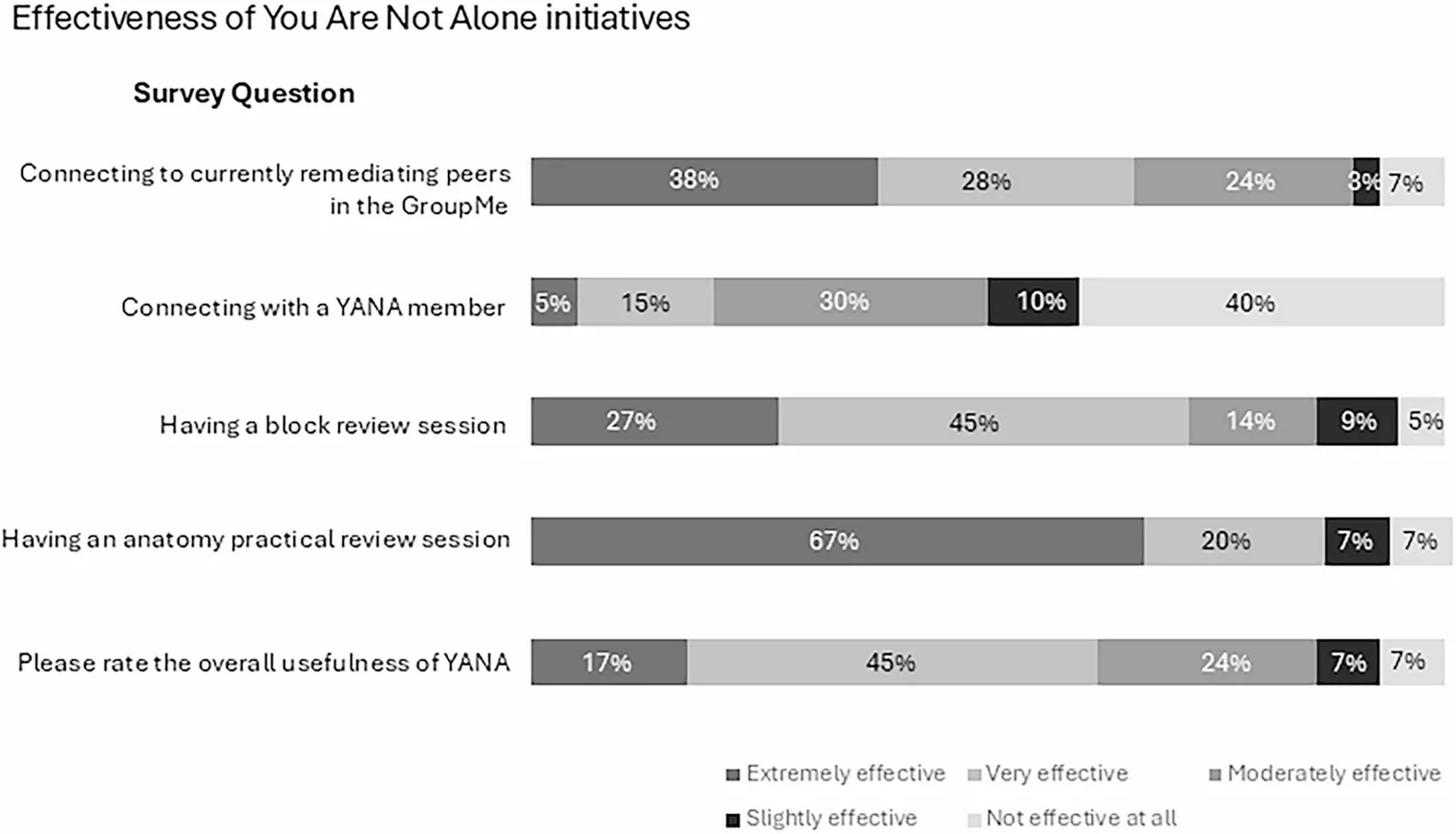
Survey results for items evaluating the perceived effectiveness of YANA. Likert scale ranged from “extremely effective” to “not effective at all.” Data reported as percent of respondents who engaged YANA. Figure created using Microsoft PowerPoint

Of the students who engaged YANA, most strongly agreed or agreed that YANA helped them feel supported (84%), less isolated (81%), and reduced their anxiety about remediating (61%) (Fig. 1). Most (67%) students strongly agreed or agreed that connecting with YANA boosted confidence of their ability to remediate, while 42% strongly agreed or agreed they would have prepared for the remediation differently if they did not engage YANA suggesting YANA’s effects extend beyond academic preparation. The majority (84%) strongly agreed or agreed they would recommend YANA to a friend. Importantly, 81% of students strongly agreed or agreed that YANA helped reframe remediation as part of the learning process (Fig. 1). Sixty-six percent of remediating students found it extremely or very effective to connect with remediating peers, while connecting with a YANA member was rated as extremely or very effective by 21% of students (Fig. 2), suggesting that meeting other remediating students was key to YANA’s success. The faculty-led review sessions YANA organized were rated extremely or very effective by 87% and 72% of students, respectively (Fig. 2). Sixty-two percent of students rated YANA as extremely or very effective overall. Fifteen respondents commented about what was most helpful about YANA represented by the following:

*YANA connecting you with other students who are remediating was by far the most helpful (arguably more helpful than anything the school did) thing for remediation.*

*I felt like it forced me out of isolation and helped me feel supported.*

*I gained friendships and support through YANA that allowed me to succeed in two remediations.*

*YANA was a great tool to help connect me to peers who also remediated and ultimately helped me create a friend group for the rest of the preclinical curriculum.*

Study limitations include the use of self-reported data, which may evoke recall bias given the time that elapsed for some respondents. The response rate was 28% and the sensitive nature of YANA’s work mandated an anonymous survey, precluding assessment of non-response bias or correlative studies; however, examination performance was not a measure of YANA’s success since most students remediate successfully anyway. A larger than expected proportion of respondents did not engage with YANA, suggesting additional work is needed to reach more students, since studies show peer support has wider benefits [11, 12]. YANA was conceived as a near-peer support group to meet a need for students to connect over shared experience, encourage each other and reframe remediation as part of the learning process and data indicate this was successful. Since YANA members are students who previously remediated it suggests that role modeling may underpin its effectiveness suggesting social learning theory as a possible framework [13, 14]. Future work will explore how YANA membership impacts the students providing near-peer support as studies have reported personal gratification among supporters [11, 12, 15, 16].

The prevalence of psychological distress among medical students is growing, suggesting that student support requires a multifaceted approach [4, 5]. Groups like YANA may complement but cannot replace college resources or professional help. YANA’s benefits are in fostering a supportive learning environment of engaged peers who assist in reframing the role of remediation in learning and manage the isolation felt by students in difficulty. YANA is a sustainable, no cost means to support students in difficulty that we believe other institutions could adopt.

## Supplementary Information

Below is the link to the electronic supplementary material.

Supplementary file1 (DOCX 15 KB)

Supplementary file2 (DOCX 20 KB)

## Data Availability

Not applicable.
