# Supplementary material for: You Are Not Alone: A Medical Student Lead, Near-Peer Program to Support Preclinical Medical Students Remediating Examinations
Source: Med Sci Educ. 2026 Sep 15;36(5):2177–81. doi: 10.1007/s40670-026-02880-8 (PMC13723629; doi:10.1007/s40670-026-02880-8)
Supplement: Supplementary file 1 [file 40670_2026_2880_MOESM1_ESM.docx]

Hello <student>,

I know you received some unwelcome news today about your performance in the <Block name> block. I want to assure you this is not an uncommon occurrence. As you know, the college has many resources available to help you remediate this block and continue successfully in the program.

One resource many students have found helpful in the past is connection with the **You Are Not Alone (YANA) group**. This is a group of M2/M3/M4 students who have remediated portions of the curriculum in the past and will offer you guidance and support as you do the same. If you would like to be connected to this group, you and others who will be remediating the <Block name> exam can do so by joining this GroupMe: <GroupMe link>.

I can also connect you with an individual from the group if you would rather not join the GroupMe. Just let me know if you prefer this.

YANA has also arranged a review for students who will be remediating. <Faculty name> has offered to host a review session for remediating students **<day, date, time>**. <Faculty name> has a slide deck for the students and will welcome questions throughout. Here is a Zoom link for that session: <Session link>.

Finally, I would love to schedule a time to meet with you to talk about how to best review your exam in preparation for the remediation and about ways you may be able to tweak your current study practices for improvement in future blocks. I’m also happy to answer any other questions you have about this process. I have good availability the rest of this week – please send me a few times that work best for you and I’ll get something scheduled for us.

Take care,
