# Supplementary material for: You Are Not Alone: A Medical Student Lead, Near-Peer Program to Support Preclinical Medical Students Remediating Examinations
Source: Med Sci Educ. 2026 Sep 15;36(5):2177–81. doi: 10.1007/s40670-026-02880-8 (PMC13723629; doi:10.1007/s40670-026-02880-8)
Supplement: Supplementary file 2 [file 40670_2026_2880_MOESM2_ESM.docx]

How did you choose to engage with YANA? (If you did not engage with YANA, please complete this question and submit).

A. I did not engage with YANA because I did not want anyone to know I was remediating
B. I did not engage with YANA because I felt I could remediate successfully on my own
C. I engaged with YANA through GroupMe
D. I engaged with YANA by talking with a member of the group privately
E. Other

Please rate the following statements based on your experiences with YANA.

1. Connecting with YANA made me feel more confident in my ability to remediate the examination.

Strongly disagree; Disagree; Agree; Strongly Agree

2. YANA made me feel supported as I prepared for the examination remediation

Strongly disagree; Disagree; Agree; Strongly Agree

3. I would have prepared for the remediation differently had I not participated in YANA.

Strongly disagree; Disagree; Agree; Strongly Agree

4. YANA helped me feel less isolated as I prepared for the examination remediation.

Strongly disagree; Disagree; Agree; Strongly Agree

5. Participating in YANA reduced the anxiety I had about examination remediation.

Strongly disagree; Disagree; Agree; Strongly Agree

6. Participating in YANA helped me realize that remediations are part of the learning process for many students.

Strongly disagree; Disagree; Agree; Strongly Agree

7. I would recommend YANA to a classmate remediating an exam in the future.

Strongly disagree; Disagree; Agree; Strongly Agree

Please rate how effective the following YANA initiatives were in helping you successfully remediate and examination.

8. Connecting to currently remediating peers through the YANA GroupMe

Not at all effective; Slightly effective; Moderately effective; Very effective; Extremely effective

9. Connecting with a YANA upperclassman mentor.

Not at all effective; Slightly effective; Moderately effective; Very effective; Extremely effective

10. Having a block review session offered for the remediated block(s).

Not at all effective; Slightly effective; Moderately effective; Very effective; Extremely effective

11. Having an anatomy practical review session offered for the remediated block(s).

Not at all effective; Slightly effective; Moderately effective; Very effective; Extremely effective

12. Please rate the overall effectiveness of YANA.

Not at all effective; Slightly effective; Moderately effective; Very effective; Extremely effective

13. Please share what was most helpful about YANA to you and why. [Optional free text]
